# Tick microbial associations at the crossroad of horizontal and vertical transmission pathways

**DOI:** 10.1186/s13071-022-05519-w

**Published:** 2022-10-21

**Authors:** Aleksandra Iwona Krawczyk, Sam Röttjers, Maria João Coimbra-Dores, Dieter Heylen, Manoj Fonville, Willem Takken, Karoline Faust, Hein Sprong

**Affiliations:** 1 Centre for Infectious Disease Control, National Institute for Public Health and the Environment, Antonie van Leeuwenhoeklaan 9, 3720 MA Bilthoven, The Netherlands; 2 Laboratory of Entomology, Wageningen University & Research, 6708PB Wageningen, The Netherlands; 3 Department of Microbiology, Immunology and Transplantation, Laboratory of Molecular Bacteriology, KU Leuven, Rega Institute for Medical Research, 3000 Leuven, Belgium; 4 Centre for Environmental and Marine Studies (CESAM), Departamento de Biologia Animal, Faculdade de Ciências, Universidade de Lisboa, Lisbon, Portugal; 5 Interuniversity Institute for Biostatistics and Statistical Bioinformatics, Hasselt University, Diepenbeek, Belgium; 6 Department of Ecology and Evolutionary Biology, Princeton University, 106A Guyot Ln, Princeton, NJ 08544 USA

**Keywords:** *Ixodes ricinus*, Microbiome, Transmission dynamics, Lyme borreliosis, Anaplasmosis, Tick-borne diseases

## Abstract

**Background:**

Microbial communities can affect disease risk by interfering with the transmission or maintenance of pathogens in blood-feeding arthropods. Here, we investigated whether bacterial communities vary between *Ixodes ricinus* nymphs which were or were not infected with horizontally transmitted human pathogens.

**Methods:**

Ticks from eight forest sites were tested for the presence of *Borrelia burgdorferi* sensu lato, *Babesia* spp., *Anaplasma phagocytophilum*, and *Neoehrlichia mikurensis* by quantitative polymerase chain reaction (qPCR), and their microbiomes were determined by 16S rRNA amplicon sequencing. Tick bacterial communities clustered poorly by pathogen infection status but better by geography. As a second approach, we analysed variation in tick microorganism community structure (in terms of species co-infection) across space using hierarchical modelling of species communities. For that, we analysed almost 14,000 nymphs, which were tested for the presence of horizontally transmitted pathogens *B. burgdorferi* s.l., *A. phagocytophilum*, and *N. mikurensis*, and the vertically transmitted tick symbionts *Rickettsia helvetica*, *Rickettsiella* spp., *Spiroplasma ixodetis*, and *Candidatus* *Midichloria mitochondrii*.

**Results:**

With the exception of *Rickettsiella* spp., all microorganisms had either significant negative (*R. helvetica* and *A. phagocytophilum*) or positive (*S. ixodetis*, *N. mikurensis*, and *B. burgdorferi* s.l.) associations with *M. mitochondrii*. Two tick symbionts, *R. helvetica* and *S. ixodetis*, were negatively associated with each other. As expected, both *B. burgdorferi* s.l. and *N. mikurensis* had a significant positive association with each other and a negative association with *A. phagocytophilum*. Although these few specific associations do not appear to have a large effect on the entire microbiome composition, they can still be relevant for tick-borne pathogen dynamics.

**Conclusions:**

Based on our results, we propose that *M. mitochondrii* alters the propensity of ticks to acquire or maintain horizontally acquired pathogens. The underlying mechanisms for some of these remarkable interactions are discussed herein and merit further investigation.

**Graphical Abstract:**

**Figure:**
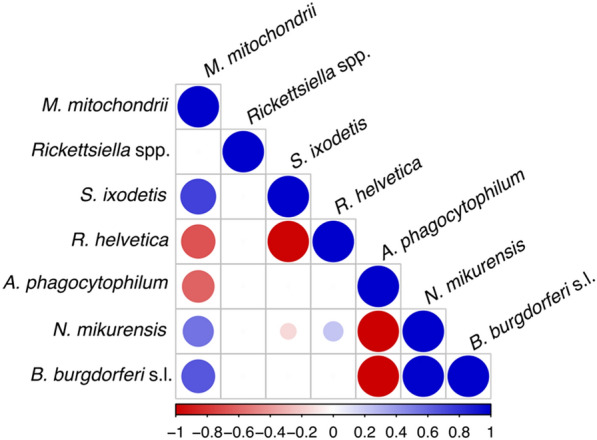
Positive and negative associations between and within horizontally and vertically transmitted symbionts

**Supplementary Information:**

The online version contains supplementary material available at 10.1186/s13071-022-05519-w.

## Background

There is increasing evidence that members of the arthropod microbiome can decrease vector-borne disease risk [1–3]. Microbes can modulate the vectorial competence of an arthropod by decreasing their susceptibility to pathogens and reducing pathogen transmission, both of which are necessary for pathogen maintenance in enzootic cycles. For instance, an *Enterobacter* bacterium isolated from wild *Anopheles gambiae* mosquitoes in Zambia confers resistance to infection with the malaria parasite in 99% of mosquitoes within the population by interfering with its development prior to the invasion of the midgut epithelium [1]. A microsporidian symbiont in another member of the *An. gambiae* complex is thought to play a similar role [4].

Like mosquitoes, ticks can transmit a plethora of pathogens causing disease in humans and domestic animals [5, 6]. Thus, an improved understanding of interactions between tick microorganisms is necessary for the development of novel strategies for controlling tick-borne diseases. A necessary first step is to understand whether and how tick microbiomes can impede or facilitate the transmission of pathogens. The microbiome of ticks consists of viruses, bacteria, and protozoa. Their relationships with ticks are often context-dependent and range from obligate mutualistic to exclusively parasitic [7–9]. In principle, all microorganisms (including tick-borne pathogens) relying on ticks for their survival are called symbionts. Here, we refer to microorganisms that have been shown to cause disease in humans and animals as pathogens.

In ticks, bacteria are the most commonly studied organisms in the microbiome-disease risk context. To date, studies have shown the mutually exclusive occurrence of pathogenic and non-pathogenic *Rickettsia* species in *Amblyomma maculatum* and *Dermacentor andersoni* ticks [10–12], as well as a negative association between the occurrence of *Rickettsia bellii* and *Anaplasma marginale* in *D. andersoni* ticks [13]. Regarding facilitative interactions, *Candidatus* Midichloria mitochondrii (hereafter *M. mitochondrii*) has been shown to be a successful colonizing partner of pathogenic *Rickettsia parkeri* in *A. maculatum* ticks [14].

In *Ixodes ricinus*, the best-studied symbionts include obligate intracellular bacteria belonging to the *Rickettsia*, *Midichloria*, *Rickettsiella*, and *Spiroplasma* genera, which are predominantly transmitted vertically from a female tick to her eggs [15]. Nevertheless, little is known about interactions between the tick microbiome members and pathogens such as *Borrelia*, *Anaplasma*, and *Neoehrlichia*. Although *Borrelia burgdorferi* sensu lato, responsible for Lyme disease, has been occasionally detected in larvae [16, 17], the aforementioned pathogens are predominantly transmitted horizontally [18–20]. In other words, ticks are born without and only acquire them as larvae or nymphs while feeding on vertebrates. The exception is *Borrelia miyamotoi*, a relapsing fever spirochete, in which vertical transmission is more efficient than horizontal [21].

The colonization of ticks by pathogens may be inhibited by vertically transmitted symbionts, which are already present in larvae. Evidence from other arthropod–pathogen systems suggests the involvement of several potential mechanisms, including direct killing, competition, and enhancement of host immune responses [22, 23]. Before describing these mechanisms, a crucial first step is to identify associations between bacterial species. For this purpose, co-infection analyses are frequently applied. However, until now, studies investigating co-infections of vertically and horizontally transmitted symbionts have reported contradictory results. For example, in *Ixodes* ticks, observed associations between *Rickettsia* and *Borrelia* have ranged from negative to positive [24–26].

At the same time, horizontally transmitted pathogens may engage in strategies to promote their replication in the presence of other microbes. Some studies have shown that *Anaplasma* and *Borrelia* may alter the tick gut microbiome to increase their colonization success by adjusting a tick antibacterial protein expression [27, 28]. However, existing evidence is limited to laboratory experiments, and it remains an outstanding question of how co-infection alters the host microbiome under natural conditions.

Here, we tested two hypotheses. The first hypothesis is that ticks infected with pathogenic bacteria such as *B. burgdorferi* s. l., possess a different bacterial microbiome than uninfected ticks or ticks infected with the pathogenic protozoan *Babesia*, bacterial *B. miyamotoi*, *Anaplasma phagocytophilum*, or *N. mikurensis*. To test this, we performed 16S rRNA amplicon sequencing on pools of *I. ricinus* nymphs infected with one of these pathogens and compared them with each other.

Importantly, *I. ricinus* is a three-host generalist tick, feeding once per life stage (larva, nymph, and adult) on various vertebrates, all of which may be amplification hosts of human pathogens [29]. Because questing nymphs feed only once as larva, the nymphal microbiome predominantly consists of vertically transmitted symbionts and, potentially, of a horizontally acquired pathogen(s).

The second hypothesis is that vertically transmitted members of the tick microbiome either facilitate or impede the acquisition and maintenance of pathogens. To test this, we used data obtained in previous studies [30, 31], where approximately 14,000 individual questing nymphs from 19 locations scattered across the Netherlands were screened for the presence of the horizontally transmitted pathogens *B. burgdorferi* s.l., *A. phagocytophilum*, and *N. mikurensis* as well as the predominantly vertically transmitted symbionts *M. mitochondrii*, *Rickettsia helvetica*, *Rickettsiella* spp. and *Spiroplasma ixodetis* [15]. Subsequently, we analysed microorganism co-infections within tick individuals using hierarchical modelling of species communities (HMSC), which tests whether microorganisms co-occur more (or less) often than by chance, given the local geographical prevalence.

## Methods

### Study sites

**Fig. 1:**
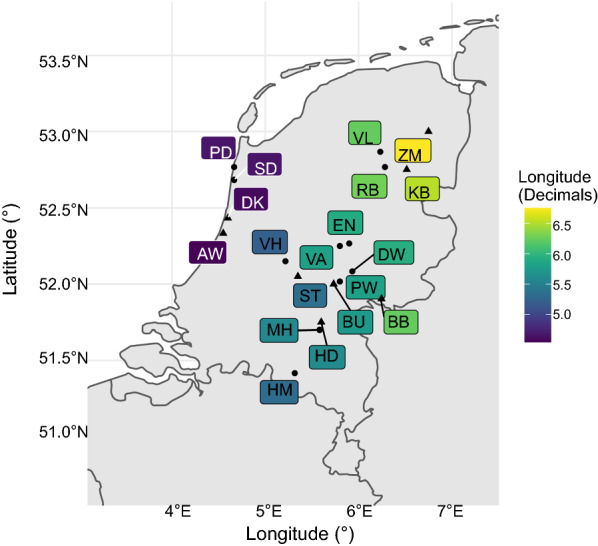
Sampling sites of *I. ricinus* in the Netherlands. Pools of nymphs from eight forest sites (triangles) were used for 16S rRNA amplicon sequencing analysis. Individual nymphs from these and 11 other forest sites (points) were tested by qPCR for the presence of tick microorganisms and used for co-infection analyses. A box marks the sampling site by two letters, and a linear colour gradient represents longitude. Full coordinates, habitat, vegetation cover, tick density, and the number of vertebrate species per location are provided in Additional file 1: Table S1

We analysed questing *I. ricinus* from eight forest sites in the Netherlands (Fig. 1). The study sites were selected based on existing knowledge of *Borrelia* genospecies prevalence, the density of ticks, vegetation profile, and vertebrate composition obtained in a previous study [31]. The full names of the sites and their coordinates are provided in Additional file 1: Table S1.

### Sample collection, DNA extraction, and pathogen detection

For the microbiome analyses, we collected a total of 7874 questing *I. ricinus* nymphs in 2016 and 2017 (Additional file 1: Table S1). All ticks were washed three times in 70% ethanol, and the DNA of individual nymphs was extracted with ammonium hydroxide as described previously [32]. The nymphs were analysed individually for the presence of tick-borne pathogens with multiplex real-time polymerase chain reaction (PCR), based on various target genes as described previously, including *B. burgdorferi* s.l. [33], *B. miyamotoi* [34], *N. mikurensis* [35], *A. phagocytophilum* [36], and *Babesia* spp.), which were designed to detect *Babesia divergens*, *B. venatorum* (formerly called EU1-3), *B. capreoli*, and *B. odocoilei* [37]. A detailed description of the quantitative PCR (qPCR) protocol is provided in Additional file 1: Table S2. Samples positive for *B. burgdorferi* s.l. were subjected to conventional PCR followed by sequencing for genotype identification [38]. After testing for tick-borne pathogens, ticks were pooled and re-extracted using the QIAGEN DNeasy Blood & Tissue Kit according to the manufacturer’s protocol (QIAGEN, Venlo, Netherlands). Pools consisted of DNA from five nymphs, which were positive for the same pathogen and negative for all the others. Sample metadata are provided in Additional file 1: Table S3, left table. Pools of nymphs negative for all pathogens were also included. For each location, a minimum of six and a maximum of 49 pools were processed (Additional file 1: Table S3, right table). Two negative controls, which underwent the same processes as tick samples including crushing, extraction, and amplification, and one company internal control were included. The DNA concentration in all samples was measured with the Qubit dsDNA HS Kit (Thermo Fisher Scientific, Ochten, Netherlands).

### 16S rRNA quantification and absolute bacterial density

In all samples, absolute bacterial density was quantified, and proportions were multiplied by a load to convert relative into absolute abundance. The quantification of total bacterial DNA load was determined by 16S rRNA qPCR as previously described [39–41]. The details on a positive control, primers, protocol, and in silico analysis can be found in Additional file 1: Table S4. It should be noted that the primers were not developed specifically for tick-associated microorganisms, and that in this study, the 16S rRNA qPCR was used in addition to other methods. The use of absolute bacterial density is a cost-effective and scalable solution for datasets of this size, since quantification methods through flow cytometry are not compatible with the sampling technique. Samples were normalized, and the normalized abundance values were scaled by the 16S rRNA qPCR loads (ng/µl) of each sample.

### Microbial profiling and taxonomic clustering

Illumina MiSeq sequencing libraries targeting the V3-V4 region of the 16S rRNA amplicon were generated and sequenced by BaseClear (Leiden, Netherlands). In short, barcoded amplicons from the V3-V4 region of 16S rRNA genes were generated using a two-step PCR. Between 10 and 25 ng of genomic (g)DNA was used as a template for the first PCR with a total volume of 50 µl using the 341F (5′-CCTACGGGNGGCWGCAG-3′) and the 785R (5′-GACTACHVGGGTATCTAATCC-3′) primers appended with Illumina adaptor sequences. Control PCR reactions were performed alongside each separate amplification without the addition of a template. PCR products were purified, and the size of the PCR products was checked on a fragment analyser (Advanced Analytical) and quantified by fluorometric analysis. Purified PCR products were used for the second PCR in combination with sample-specific barcoded primers (Nextera XT index kit, Illumina). Subsequently, PCR products were purified, checked on the fragment analyser (Advanced Analytical) and quantified, followed by multiplexing, clustering, and sequencing on an Illumina MiSeq with the paired-end (2×) 300-base-pair (bp) protocol and indexing. The sequencing run was analysed with the Illumina CASAVA pipeline (v1.8.3) with demultiplexing based on sample-specific barcodes. The raw sequencing data produced were processed by removing the sequence reads of inadequate quality (only “passing filter” reads were selected) and discarding reads containing adaptor sequences or PhiX control using an in-house filtering protocol. A quality assessment on the remaining reads was performed using the FastQC version 0.10.0 quality control tool. Sequenced reads were imported to the QIAGEN CLC Genomics Workbench 10.0.1 supplemented with CLC Microbial Genomics Module 3.6.1 (www.clcbio.com). Overlapping pairs of raw reads were merged into single longer reads and trimmed with a quality score limit of 0.05 and two ambiguous nucleotides. At this stage, primer sequences were trimmed. Subsequently, reads were fixed-length trimmed (~ 400 bp). To identify operational taxonomic units (OTUs), reads were clustered using the SILVA 16S version 128 reference database with 97% identity as the clustering criterion. Chimeras were removed with a built-in tool in the CLC Genomics Workbench.

### Microbiome analyses

All analyses were carried out in R 3.6.3 [42]. We used the R package *vegan* (version 2.5-6) for ordination [43]. A principal coordinate analysis (PCoA) was carried out using Bray–Curtis dissimilarities. Results were visualized with ggplot2 (version 3.3.2; [44]).

Additionally, we carried out permutational multivariate analysis of variance (PERMANOVA) [45] with the *adonis* function from R package *vegan* to assess whether pathogen presence significantly affected community variation*.* We tested for multivariate dispersion through the *betadisper* function.

### Co-infection analyses

To investigate co-infections of tick microorganisms, we utilized the qPCR data on the infection prevalence of tick symbionts and pathogens generated in previous studies [30, 31, 46]. Briefly, questing nymphs of *I. ricinus* were collected from 19 forest sites in the Netherlands in 2013 and 2014 (Fig. 1). A total of 13,968 individual nymphs of *I. ricinus* were screened for *S. ixodetis*, *R. helvetica*, *Rickettsiella* spp., *M. mitochondrii*, *A. phagocytophilum*, *N. mikurensis*, and *B. burgdorferi* s.l. *Borrelia miyamatoi* and *Babesia* spp. data were not included in further investigations due to their low prevalence, which did not allow for performing statistical analyses with confidence.

In a recent study, we found geographical differences in tick microbial community structure [30]. Therefore, to explore non-random residual associations (co-infections) of microorganisms within individual *I. ricinus* ticks while controlling for confounding factors associated with the between-individual and spatial variation, we used a recently developed statistical approach, HMSC [47]. HMSC is a generalized framework based on a hierarchical joint species distribution approach (JSDM) [48]. In the context of our study, JSDM considers information on the presence/absence of many species simultaneously, at the community level, and incorporates the effects of environmental factors and interspecific interactions on species incidence into a single model, which allows us to estimate species co-occurrence patterns (e.g. [49, 50]). An advantage of the HMSC methodology is that it allows the consideration of multiple hierarchical levels of the study design.

The R package Hmsc [51] was used to build the model. We included the tick’s identity as a sampling unit, which was nested as a random effect within the location where the tick was collected. We fitted a probit model to presence/absence data to ensure successful convergence. We sampled the posterior distribution with three Markov chain Monte Carlo (MCMC) chains, each with 1500 samples, thin 1000 and transient 750,000.

We then examined MCMC convergence by assessing the effective size of the posterior sample and Gelman and Rubin's [52] convergence diagnostic (potential scale reduction factor; PSRF) with the *gelman.diag* function. We performed variance portioning with the *computeVariancePartitioning* function to investigate which random effect was most responsible for the observed variation in the prevalence of individual microorganisms. Lastly, to compute the microorganism association matrices associated with each random level, we used the *computeAssociations* function. The *corrplot* function from the corrplot package was used to plot only those associations for which the posterior probability of being negative or positive was at least 0.95 [53].

## Results

### Microbial profiling and taxonomic clustering

A total of 165 out of 166 processed samples successfully generated 6,590,988 raw reads on an Illumina MiSeq flow cell. One sample was excluded because it had an extremely low DNA load, which resulted in no amplification. A total of 4,454,814 reads were assigned taxonomy. A total of 184,682 unique reads were clustered into 8818 OTUs (Additional file 1: Table S5). However, 1966 OTUs, which accounted for 0.7% of all reads, could not be assigned to any known taxa and were discarded from further analyses. The top 10 most abundant genera included *Borrelia*, *Midichloria*, *Neoehrlichia*, *Methylobacterium*, *Mycobacterium*, *Pseudomonas*, *Rickettsia*, *Rickettsiella*, *Sphingomonas*, and *Wolbachia* (Additional file 2: Figure S1). Despite being the most abundant taxa in some samples, *Wolbachia* was not considered an *I. ricinus* tick symbiont, and we did not include it in the further qPCR analysis. Its origin in our tick samples was due to the presence of endoparasitoid *Ixodiphagus hookeri* eggs [54, 55].

Two types of negative controls were utilized: two extractions without template DNA and one only containing sequencing reagents. The PCoA showed that all three negative controls were highly similar, indicating that any errors introduced through reagent contamination were preserved (Additional file 2: Figure S2). The ordination also demonstrated that tick samples did not neatly cluster away from the negative controls. This lack of a distinction from the negative controls is likely attributable to low bacterial abundance in some samples and a high abundance of *Pseudomonas*, a known contaminant that was highly abundant in the negative controls.

**Fig. 2:**
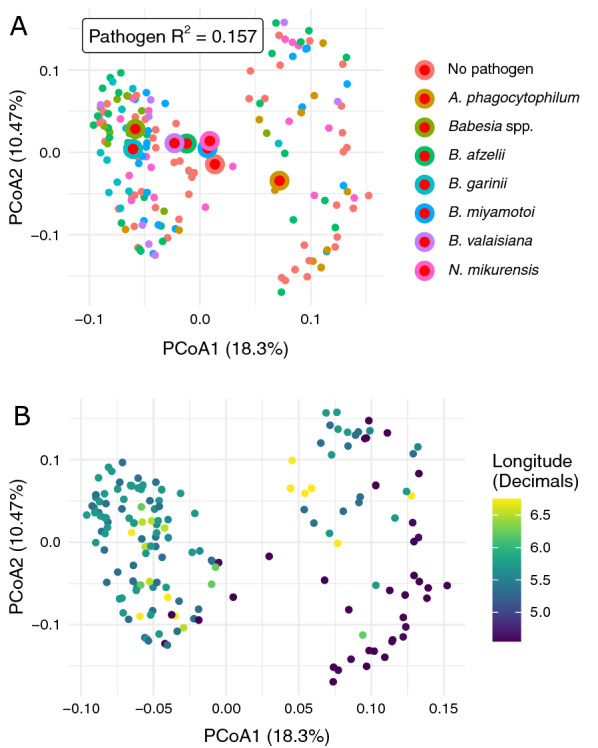
Principal coordinate analysis (PCoA) of microbial abundance scaled by 16S rRNA load. **A** Ticks were screened for the presence of specific pathogens, pooled, and then sequenced. The colour of samples indicates the detected pathogen, with the red dots representing the centroid. The *R*^2^ for pathogens was calculated using PERMANOVA. **B** PCoA coloured by longitude of sample locations

The PCoA did not convincingly show that community composition was structured by pathogen-specific pools, or separated along the pools with or without pathogens (Fig. 2A). Instead, the PCoA suggested that samples were mostly separated by the geographical region, as indicated by longitude, of tick collection (Fig. 2B). Therefore, we tested with PERMANOVA whether communities were significantly different for ticks screened positively for different pathogens. Pathogen identity had a significant yet weak relationship with sample composition (*R*^2^ of 0.157, *P* = 0.001). However, significant overdispersion (*P* < 0.001) indicates that the *P*-values may be overinflated. Although the results suggest that microbiome composition was significantly different across samples with different pathogens, the *R*^2^ demonstrates that most variation could not be attributed to a pathogen status.

### Co-infection of vertically and horizontally transmitted symbionts

Data on the prevalence of tick microorganisms in questing nymphs generated in previous studies were analysed for co-infections [30, 31, 46]. Briefly, 13,967 questing nymphs from 19 forest sites were tested with qPCR for the presence of *S. ixodetis*, *R. helvetica*, *Rickettsiella* spp., *M. mitochondrii*, *A. phagocytophilum*, *N. mikurensis*, and *B. burgdorferi* s.l. All microorganisms were present in all locations; however, infection rates varied strongly among the bacterial species and locations. The prevalence of the tick microorganisms per location and region is provided in Table S6, as well as in Krawczyk et al. [30] and Takumi et al. [31].

A total of 1732 (12.4%) tested questing nymphs were free of any of the seven studied microorganisms, and 12,225 (87.6%) nymphs were infected with one or more. The exact binomial test showed that infections with at least one, two, three, or four tick microorganisms were significantly less frequent than expected (*P* < 0.001 in all cases), and infections with at least five, six, or seven microorganisms occurred as often as expected (Additional file 1: Table S7). A single infection was detected in 4791 nymphs, a double infection in 4992 nymphs, a triple infection in 2177 nymphs, and a quadruple infection in 307 nymphs. A total of 27 nymphs were infected with five, one with six, and none with seven microorganisms, showing that co-infections are not randomly distributed in tick populations.

To investigate co-infections of tick microorganisms, we constructed an HMSC model in which we included the tick’s identity as a sampling unit, which was nested as a random effect within the location where the tick was collected. The MCMC convergence of the model was satisfactory: the potential scale reduction factors for the β-parameters were 1.056 on average (max = 1.211). Variance partitioning showed that the tick’s identity explained a substantial proportion of the observed variation in the prevalence of *M. mitochondrii* (80.0%), *S. ixodetis* (80.1%), *N. mikurensis* (70.6%), and *B. burgdorferi* s.l. (84.7%). Location explained variation in the prevalence of *Rickettsiella* spp. (99.6%), *R. helvetica* (84.3%), and *A. phagocytophilum* (61.2%).

**Fig. 3:**
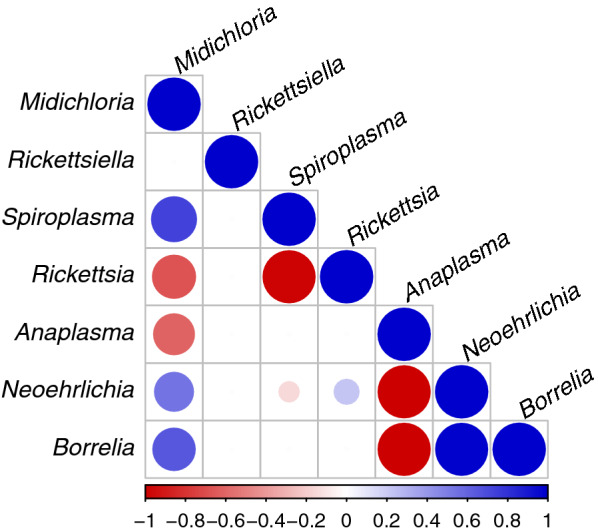
Heatmap of tick microorganism species-to-species associations (co-infections) in ticks detected based on the HMSC model with the tick’s identity as a sampling unit, after controlling for the location where the tick was collected. Blue and red colours show parameters that are estimated to be positive and negative, respectively, with at least 0.95 posterior probability. Larger and darker circles indicate stronger associations. Details on all associations are provided in Additional file 1: Table S8

Although all possible combinations of two microorganisms were found in nymphs, some combinations occurred significantly more or less often than by chance. The HMSC model showed that all microorganisms but *Rickettsiella* spp. had either significant negative (*R. helvetica* and *A. phagocytophilum*) or positive (*S. ixodetis*, *N. mikurensis*, and *B. burgdorferi* s.l.) associations with *M. mitochondrii* (Fig. 3). *Borrelia burgdorferi* s.l. and *N. mikurensis* had a significant positive association with each other and a negative association with *A. phagocytophilum* (Fig. 3)*. Neoehrlichia mikurensis* also had a significant negative association with *S. ixodetis* and a positive association with *R. helvetica* (Fig. 3)*.* Lastly, two tick symbionts, *S. ixodetis* and *R. helvetica*, had a significant negative association with each other (Fig. 3).

## Discussion

This study detected significant negative and positive associations between tick microorganisms known to utilize different transmission routes. Populations of ticks carrying *M. mitochondrii* were more likely to carry another tick symbiont, *S. ixodetis,* and the pathogens *B. burgdorferi* s.l. and *N. mikurensis*, than were ticks without *M. mitochondrii* (Fig. 3). Nevertheless, we did not observe differences in the bacterial microbiome of ticks infected with distinct horizontally acquired pathogens such as *Borrelia afzelii*, *Borrelia garinii*, *Borrelia valaisiana*, *B. miyamotoi*, *Babesia*, *A. phagocytophilum*, and *N. mikurensis* (Fig. 2).

### Microbiome of *I. ricinus*

We did not detect differences between the bacterial microbiome of ticks with and without pathogens (Fig. 2). As the presence of a pathogen in nymphs indicates that it fed on a specific vertebrate host as a larva, our findings imply that the vertebrate host has little to no effect on the tick bacterial community. Our results are in line with previous findings by Hawlena et al. [56], but they contrast with a study by Swei and Kwan [57], who found differences in the microbiome of *I. pacificus* ticks feeding on lizards and mice. We are not aware of any of the nymphs studied here feeding on lizards, which are relatively uncommon tick hosts in our study areas [29]. Nevertheless, we cannot rule out the potential influence of reptiles on the tick microbiome. Non-avian reptiles, as exothermic vertebrates, have less stringent requirements for regulating blood biochemical properties, and their blood may have vastly different osmotic pressure and pH compared with that of mammals and birds [58]. While we could not investigate such effects in this study, this could lead to the absence of host effects compared with the work by Swei and Kwan [57].

A possible explanation for our finding could be that ticks are genetically equipped to resist opportunistic bacteria, such as host skin commensals. Hayes et al. [59] discovered that *I. scapularis* horizontally acquired an antimicrobial toxin gene from bacteria. The gene encodes lytic cell wall-degrading enzymes delivered to the host bite site via saliva, which is responsible for selectively killing the skin-associated bacteria of the vertebrate. Interestingly, the enzymes had no intrinsic lytic activity against *B. burgdorferi* [59]. Therefore, pathogens may still affect the tick gut microbiome to facilitate their colonization, as previously observed in laboratory experiments [27, 28]. In future work, investigating individual organs deriving from field-collected ticks may extend our understanding of microbiome–pathogen interactions.

Beta diversity of the bacterial communities varied by geographical region, as indicated by longitude, which is in accord with previous studies [30, 60–62]. In our earlier study [30], we found a heterogeneous distribution of tick symbionts across space, with *Rickettsia* abundance being the most likely driver of the differences in observed bacterial communities in ticks.

Therefore, in our pairwise analysis exploring the co-infections between tick microorganisms in individual nymphs, we used a model controlling for location as well as tick identity.

### Associations of vertically transmitted symbionts

We detected significant negative associations of *R. helvetica* with two other tick symbionts, *M. mitochondrii* and *S. ixodetis*, which were positively associated with each other (Fig. 3). Interestingly, in previous studies on the microbiome of *I. ricinus,* Aivelo et al. [63] and Lejal et al. [64] also detected a negative association between *Rickettsia* sp. and *Spiroplasma*. The former study did not investigate the association of *Rickettsia* sp. with *M. mitochondrii*, which was present in all samples, while the latter found a positive association.

It is possible that ticks cannot simultaneously maintain two symbiont species by vertical transmission, which may affect their distribution in tick populations. The phenomenon of interference between two vertically transmitted symbionts in hard ticks (Ixodidae) has been described in previous studies [10, 12, 65, 66]. Primary infection with one *Rickettsia* species has been suggested to block (by an unknown mechanism) transovarial transmission of a second *Rickettsia* species. However, to our knowledge, this so-called rickettsial interference has never been related to any other tick symbiont genus. If this interference between *R. helvetica* and *S. ixodetis* exists, to persist in a tick population, *S. ixodetis* would have to occasionally utilize horizontal transmission, which has been suggested but not undisputedly proven [67, 68].

The mutual exclusion among symbionts may also indicate that the roles which they play in ticks overlap and that a double infection comes with fitness costs without providing sufficient increases in fitness. For instance, in *Acyrthosiphon pisum*, both *Serratia symbiotica* and *Hamiltonella defensa* provide resistance to parasitoids. Although a double infection provided increased resistance in the laboratory, it was rarely observed under natural conditions [69].

The positive association between *M. mitochondrii* and *S. ixodetis* associations imply that these pairs of symbionts serve complementary functions and/or exhibit different tissue tropism in ticks. Different host tissues may constitute distinct microhabitats and be nutritionally favourable, immunotolerant, and easy to colonize for some but not for other microorganisms [70].

### Associations of horizontally with vertically transmitted microorganisms

Our analyses showed that the most prevalent tick symbiont, *M. mitochondrii*, was positively associated with the pathogens *B. burgdorferi* s.l. and *N. mikurensis*, and negatively associated with the pathogen *A. phagocytophilum* (Fig. 3). Although these specific associations have not been reported previously, *M. mitochondrii* has been shown to be a successful colonizing partner of pathogenic *R. parkeri* in *A. maculatum* ticks [14].

It is possible that *M. mitochondrii*, which infects female ticks more often than males [71], enhances the ticks’ probability of becoming infected with zoonotic pathogens. Generally, immature female *I. ricinus* are larger and take lengthier and more extensive blood meals than males [72, 73], which might facilitate the acquisition of a pathogen from a host.

Additionally, the observed positive associations of the *M. mitochondrii* symbionts with *B. burgdorferi* s.l. and *N. mikurensis* could be indirect in nature and arise from this symbiont, providing fitness benefits to ticks. It is suggested that *M. mitochondrii* increases tick survival by supplying essential nutrients, which enhances reproductive fitness, in addition to benefiting energy production, maintenance of homeostasis and water balance, and antioxidant defence [74].

However, in our study, it appears that this facilitation occurs only for *B. burgdorferi* s.l. and *N. mikurensis*, which circulate among small mammals such as rodents and birds [75–78], and not for *A. phagocytophilum*. The acquisition of *A. phagocytophilum* occurs mostly while ticks feed on deer [79], a host fed upon by nymphal rather than larval tick stages [80]. Therefore, it is possible that at the adult stage, where ticks had a chance to feed twice (once as a larva and once as a nymph), a positive association between *A. phagocytophilum* and *M. mitochondrii* could be detected.

### Associations of horizontally transmitted pathogens

Different species of horizontally transmitted symbionts can be acquired from either a single host species or multiple species of vertebrate hosts [18, 77–79, 81], which is often reflected in their co-infection patterns in nymphs that fed only once as larvae. For instance, we observed a strong positive association between *B. burgdorferi* s.l. and *N. mikurensis*, which we expected given that both *N. mikurensis* and *B. afzelii* (the most prevalent genospecies of *B. burgdorferi* s.l. in *I. ricinus* in the Netherlands [82]) are amplified by rodents, which are often simultaneously infected with both microorganisms [68, 75, 76].

*Anaplasma phagocytophilum* may be acquired by *I. ricinus* from various vertebrate species, but it is most commonly acquired from roe deer [79]. Therefore, the significant negative association of *A. phagocytophilum* with *B. burgdorferi* s.l. and *N. mikurensis* pathogens implies that nymphs that fed on deer had no chance to acquire rodent-borne pathogens [68].

## Conclusions

Although here a few specific associations did not have a large effect on the composition of the entire microbiome, they can still be relevant for tick-borne pathogen dynamics. We observed that tick symbionts are heterogeneously distributed across the Dutch tick population and revealed a novel pattern of associations. Although the underlying mechanisms are unknown, they are important for understanding the role of vertically transmitted symbionts in the control of tick-borne diseases.

For instance, our findings imply that ticks carrying *M. mitochondrii* contribute more to the transmission cycle and the acarological risk of Lyme borreliosis and neoehrlichiosis than ticks lacking *M. mitochondrii.* Future studies on microorganism interference should investigate the dynamics of symbionts through the ontogeny of pre-infected ticks in comparison with non-infected ticks, applying bacterial quantification methods and identifying the microbiomes of ticks during different life events such as moulting, questing, and feeding.

## Supplementary Information

**Additional file 1: Table S1.** Details on studied forest sites. **Table S2.** The qPCR protocol for detection of tick symbionts and pathogens. **Table S3.** Bacterial loads of samples for the 16S rRNA sequencing; sample scheme. **Table S4.** Details on a positive control, primers, protocol, and in silico analysis used in the 16S rRNA quantification analyses. **Table S5.** OTU table. **Table S6.** Prevalence of veritcally and horizontally transmitted symbionts and pathogens per location. **Table S7.** Expected and observed co-infections in questing nymphs and results of the exact binomial test. **Table S8.** Tick microorganism species-to-species associations (co-infections) in ticks.

**Additional file 2: Figure S1.** Abundance of the most abundant taxa, separated by quartiles of absolute bacterial density (16S rRNA content in ng/µL). **Figure S2.** Principal coordinate analysis of Bray–Curtis dissimilarities for tick microbiomes compared to blanks.

## Data Availability

The datasets used and/or analysed during the current study are available in the supplementary data files and from the corresponding author on reasonable request.
