# Supplementary material for: Tick microbial associations at the crossroad of horizontal and vertical transmission pathways
Source: Parasit Vectors. 2022 Oct 21;15:380. doi: 10.1186/s13071-022-05519-w (PMC9585727; doi:10.1186/s13071-022-05519-w)
Supplement: Supplementary file 2 — Additional file 2: Figure S1. Abundance of the most abundant taxa, separated by quartiles of absolute bacterial density (16S rRNA content in ng/µL). Figure S2. Principal coordinate analysis of Bray–Curtis dissimilarities for tick microbiomes compared to blanks. [file 13071_2022_5519_MOESM2_ESM.docx]

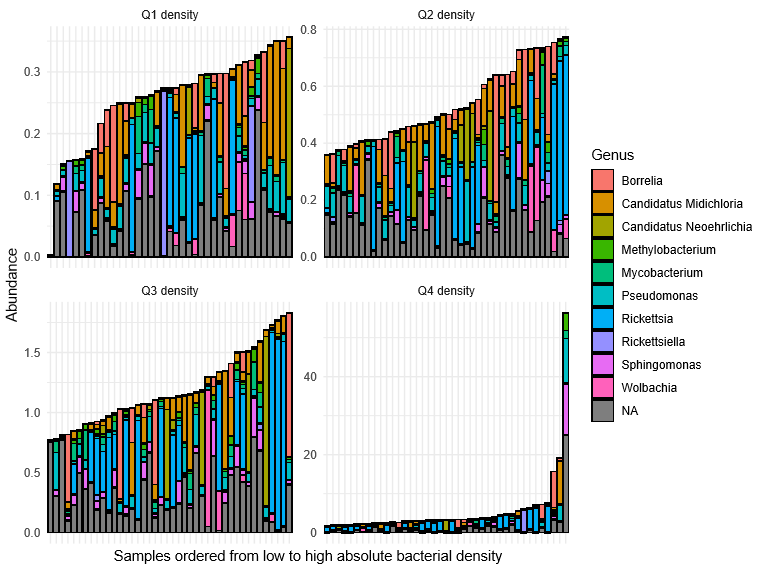


**Figure S1.** Abundances of the most abundant taxa, separated by quartiles of absolute bacterial density (16S rRNA content in ng/µL). The remaining taxa were binned in the synthetic ‘Other’ taxon. All abundances are scaled by the bacterial density. The genus Pseudomonas was also highly abundant in negative controls.


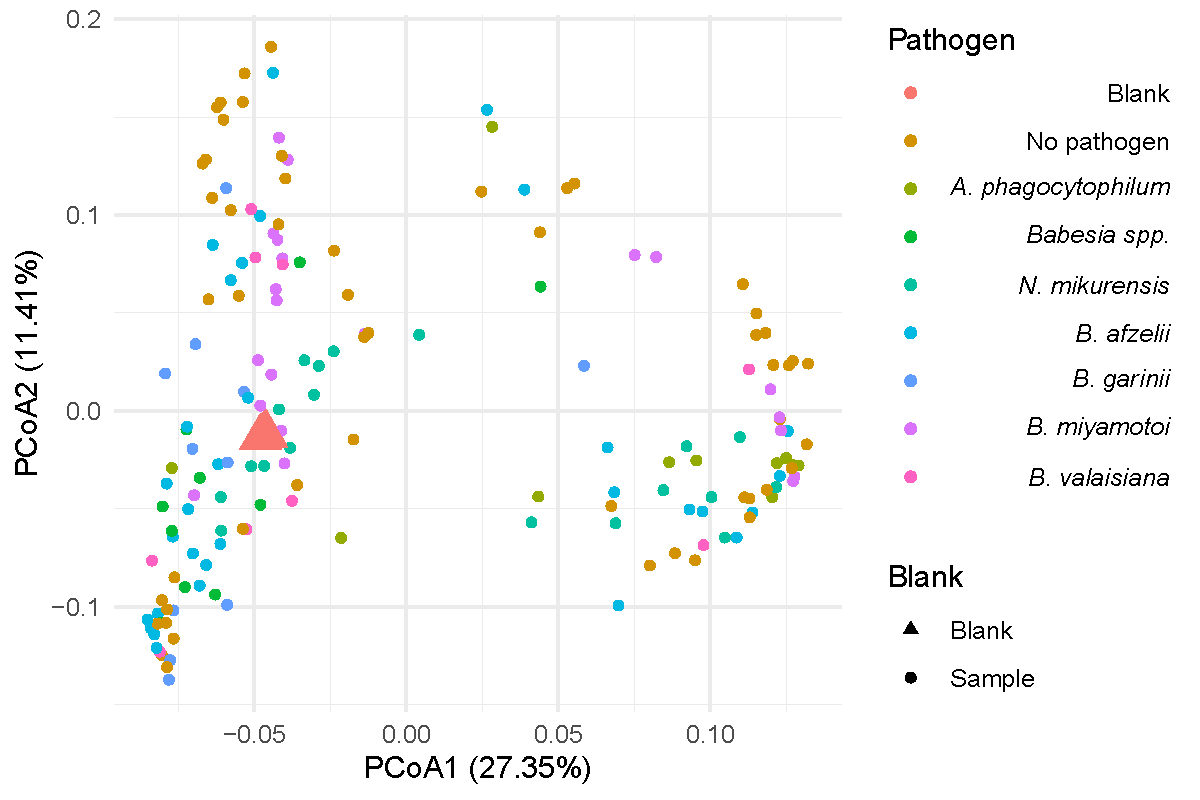


**Figure S2.** Principal Coordinate Analysis of Bray-Curtis dissimilarities for tick microbiomes compared to blanks. Counts were not corrected for 16S rRNA load. Individual ticks were screened for pathogens, pooled per pathogen, and subsequently subjected to microbiome analysis. Each sample colour reflects a pathogen. Three negative controls (blanks) are shown as a red triangle and group with many tick samples due to their similarity.
